# Using latent class cluster analysis to screen high risk clusters of birth defects between 2009 and 2013 in Northwest China

**DOI:** 10.1038/s41598-017-07076-0

**Published:** 2017-07-31

**Authors:** Leilei Pei, Lingxia Zeng, Yaling Zhao, Duolao Wang, Hong Yan

**Affiliations:** 1 Department of Epidemiology and Health Statistics, School of Public Health, Xi’an Jiaotong University Health Science Center, Xi’an, Shaanxi 710061 P.R. China; 2 Biostatistics Unit, Department of Clinical Sciences, Liverpool School of Tropical Medicine, Pembroke Place, Liverpool, L3 5QA UK

## Abstract

In the study, we aimed to explore the synergistic effects of multiple risk factors on birth defects, and examine temporal trend of the synergistic effects over time. Two cross-sectional surveys conducted in 2009 and 2013 were merged and then latent class cluster analysis and generalized linear Poisson model were used. A total of 9085 and 29094 young children born within the last three years and their mothers were enrolled in 2009 and 2013 respectively. Three latent maternal exposure clusters were determined: a high-risk, a moderate-risk, and a low-risk cluster (88.97%, 1.49%, 9.54% in 2009 and 82.42%, 3.39%, 14.19% in 2013). The synthetic effects of maternal exposure to multiple risk factors could increase the risk of overall birth defects and cardiovascular system malformation among live births, and this risk is significantly higher in high-risk cluster than that in low-risk cluster. After adjusting for confounding factors using a generalized linear Poisson model, in high-risk cluster the prevalence of nervous system malformation decreased by approximately 2.71%, and the proportion of cardiovascular system malformation rose by 0.92% from 2009 to 2013. The Chinese government should make great efforts to provide primary prevention for those on high-risk cluster as a priority target population.

## Introduction

All over the world, birth defects (BD) defined as a series of structural, functional and metabolic disorders, affect an estimated 3% of the 134 million annual births and result in about 632,000 deaths per year in 2013^1, 2^. Researches showed that the type with the greatest numbers of deaths are congenital heart disease, followed by neural tube defects^2^. The etiology of birth defects has been widely discussed but is not yet fully clarified. Numerous studies have confirmed that fewer birth defects could be attributed to a single factor, but more are generally caused by several interrelated factors, including genetics, chemicals, physical and biological issues and maternal elements^3–9^. Although it is important to tease out which risk factors, if any, contribute to the development of these birth defects, humans live or work in the environment that contain many interrelated risk factors, and thus examining the synthetic effects of all possible risk factors and screening high risk groups may also aid in prevention. Latent class cluster analysis (LCCA) is one powerful approach, which can help to address the complexity of birth defects-related factor patterning and to capture the synthetic effects. Unlike the traditional single factors approaches, LCCA aims to examine the interrelationships among multiple risk factors and classify individuals into mutually exclusive risk patterns such that variation in risk factors is maximized across different risk patterns while individuals within risk patterns have similar exposure^9, 10^. However, so far, there are few researches that used LCCA to explore the synergistic effects of multiple risk factors on birth defects, and examine temporal trend of the synergistic effects over time.

China is one of the countries with high incidence of BD, with the estimated 4–6% of Chinese newborns affected by birth defects every year^10^. Due to lower socioeconomic status and inadequate health facilities compared to other regions of China, Northwestern China may face a more severe challenge of birth defects. According to the sixth census (2010) data in China, Shaanxi province of Northwest China had a population of approximately 37 million, accounting for nearly half of the population in Northwest China. In our recent studies, it is clear that the prevalence of BD in Shaanxi province is much higher than the level of other regions of China^5^.

In Northwest China, to the best of knowledge, how to evaluate the synthetic effects of the risk factors related to birth defects and whether the synthetic effects of these factors are characterized by increasing, decreasing, or unchanging trend over time, are not known but are important for prevention and control of birth defects. Therefore, to address these aforementioned research questions, the aims of our present study were twofold: 1) to extract different latent maternal exposure clusters using latent class cluster analysis (LCCA) based on overall risk factors in 2009 and 2013 in Shaanxi province, and to explore the association between latent clusters and birth defects; 2) to evaluate the change in the prevalence of birth defects among latent clusters in Shaanxi province for the 5-year period during 2009–2013.

## Methods

### Study design and participants

In 2009 and 2013, two large cross-sectional population-based epidemiological surveys of birth defects among young children was conducted respectively in Shaanxi province, Northwest China. The target population of the two study were young children born within the last three years and their mothers. Considering the urban-rural disparity in social density and fertility rates of population in the whole province, a stratified multi-stage sampling method was adopted to determine the sampling units. Within China’s rural areas, the 3-level administrative structure consists of county, township, and village. Independent of rural areas, districts, streets and communities in urban areas are another different three-level administrative structure. In 2009, six counties in rural areas and six districts in urban areas were randomly determined throughout the province, while the 20 counties and 10 districts were randomly selected in 2013. In 2009, 6 villages/communities each from 6 townships/streets were sampled randomly from each selected counties. Similarly in the rural areas in 2013, from each selected counties, 6 villages each from 6 townships were sampled randomly. However, in the urban areas in 2013, from each chosen districts, 6 communities in each of 3 streets were selected randomly for sampling. Thirty babies born within the last three years and their mothers in each sampled village in 2009, and, 60 in each chosen community in 2013 were selected using a random sampling method. Finally, we expect to recruit a total of 16200 and 32400 participants respectively in 2009 and 2013. However, 2927 and 2374 subjects of the randomly sampled population declined to participate in the 2009 and 2013 study (response rate: 81.9% and 92.7% respectively). Therefore, approximately 13273 and 30026 participants were eventually obtained in 2009 and 2013 survey.

### Data collection

Data were collected at the local village clinics and community health service centers in 2009 and 2013 respectively. Ten investigation teams were established for field surveys and data collection. Each team consisted of 10 to 12 investigators and a supervisor from Xi’an Jiaotong University Health Science Center and a pediatric doctor from a local maternal and child health hospital. All fieldworkers were trained to standardize questionnaire administration, including lectures and practice in the field at least one month before commencement of the survey. During the survey, all fieldworkers were closely monitored by their supervisors and randomly examined. Once any errors and/or missing values were detected, subjects were required to be re-interviewed. Most important of all, our work was strongly supported by the local hospitals and health administrative departments as well as the Ministry of Health in Shaanxi province.

Only after obtaining written informed consent from all participants or their legal guardians, a face-to-face interview was performed by the field investigators using a precoded structured questionnaire, including birth defects, socio-demographic characteristics, reproductive history, family history, environmental risk factors, maternal illness during pregnancy and maternal drug use during pregnancy. Medical records at local hospitals including prenatal diagnostic test results, clinic diagnostic results, findings of physical examination, ultrasound imaging reports and medical history were used as final diagnosis references for birth defects. This study was reviewed and approved by the ethics committee of Xi’an Jiaotong University Health Science Center. All methods in the study were performed in accordance with the relevant guidelines and regulations.

### Study variables

According to the International Classification of Diseases, 10th Revision (ICD-10), birth defects were classified into eleven different categories, of which nervous system (codes Q00–Q07) and cardiovascular system (codes Q20–Q28) malformation were chosen as the main outcome variables in the study. The numbers of the whole birth defects, nervous system malformation and cardiovascular system malformation were the main outcome variables in generalized linear Poisson model. Socio-demographic characteristics included one continuous variable (parity) and six categorical variables (gender, maternal age, area, maternal education level, household economic level and residence during the pregnancy). The Demographic and Health Survey household wealth index (HWI)^11^ was used to assess the household economic level of the participants. After an HWI was calculated based on the principal component analysis of 5 variables representing family economic level (housing conditions, type of vehicle, income resources, and type and number of household appliances), the SES of the participants was divided into thirds: low, medium, and high (poor, middle-income, rich, respectively). For data dimensionality reduction, in our study, all possible risk factors for birth defects were clustered into these seven different indicator variables, including no folic acid supplementation, genetic factor, maternal illness, adverse pregnancy outcomes, drugs use, environmental risk factors, and unhealthy lifestyle during pregnancy. The values ranging from 0 to N were obtained by summing the number of risk factors in each indicator variable as total risk factors score (Table 1).

**Table 1:** The definition of latent profile analysis indicators*.

Indicator variables	Risk factors	Min	Max
No folic acid supplementation	No folic acid supplementation from 3 months before to 3 months after conception	0	1
Genetic factor	Parental consanguinity	0	3
	Birth defects in immediate family members		
	Consanguinity in immediate family members		
Maternal illness	Fever or Cold	0	8
	Anemia		
	Diabetes		
	Hepatitis		
	Thyroid disease		
	Pregnancy-induced hypertension syndrome		
	Depression		
	Others		
Adverse pregnancy outcomes	Preterm	0	4
	Stillbirth		
	Abortion		
	Birth defects		
Drugs use	Antibiotic	0	4
	Contraceptive		
	Antidepressant		
	Others		
Environmental risk factors	Pathogenic Microorganism	0	6
	Noise		
	Hot and humid exposure		
	X-ray		
	Heavy metal pollution		
	Industrial dust		
Maternal unhealthy lifestyle	Periconceptional smoking	0	5
	Periconceptional passive smoking		
	Periconceptional drinking		
	Periconceptional tea consumption		
	Periconceptional coffee consumption		

^*^All risk factors were transformed into 0/1 variables, and then were summed as a total indicator variable.

### Statistical analysis

In this study, we merged the two 2009 and 2013 databases into a large one for pooled analysis. First, latent class cluster analysis was performed for the seven risk factor variables all of which had been standardized before analysis. LCCA refers to a collection of statistical approaches in which individuals are classified into unobserved subpopulations represented by a categorical latent variable which is not observed and must be inferred from the data. Indicator variables used to determine latent classes can be continuous, censored, binary, ordered/unordered categorical counts, or combinations of these variable types^12^. This study was undertaken on continuous indicators, assuming multivariate normal distribution within latent classes. The model could be indicated as following:

1 $${\rm{f}}({{\bf{x}}}_{{\boldsymbol{i}}})={\sum }_{j=1}^{K}{\eta }_{j}\,{f}_{j}({x}_{i}|{\mu }_{j},{{\rm{\Sigma }}}_{j})={\sum }_{j=1}^{K}{\eta }_{j}{\prod }_{i=1}^{p}\frac{1}{\sqrt{2\pi {\sigma }_{ij}^{2}}}\exp (\frac{-({x}_{i}-{\mu }_{ij})}{{\sigma }_{ij}^{2}})$$

The equation assumes that *x*_*i*_ is the observed response to variable *i*, *µ*_*ij*_ and ${\sigma }_{ij}^{2}$ are the mean and variance for variable *i* for an individual from class *j*. The proportion of persons in each of the classes is denoted by *η*_*j*_, and every individual probabilities of the membership in all of the latent classes could be estimated (when summed they equal 1), reflecting the varying degrees of certainty and precision of classification. In this study, means (*µ*_*ij*_) and proportion of persons in each of the classes (*η*_*j*_) would be estimated. According to Bayes’ theorem, in the meanwhile, the posterior probability of assigning respondents to the *j* class, could be obtained and expressed as follows:

2 $${\rm{P}}(j|{x}_{i})=\frac{{\eta }_{j}{f}_{j}({x}_{i}|{\mu }_{j},{{\rm{\Sigma }}}_{j})}{{\sum }_{j=1}^{K}{\eta }_{j}{f}_{j}({x}_{i}|{\mu }_{j},{{\rm{\Sigma }}}_{j})}$$

The models with different numbers of classes are compared using information criteria (IC)-based fit statistics such as Akaike Information Criteria (AIC), Bayesian Information Criteria (BIC), Sample-size-adjusted (SSA)-BIC. Lower values on these fit statistics indicate better model fit. Entropy is a type of statistic that assesses the accuracy with which models classify individuals into their most likely class, and can range from 0 to 1, with higher scores representing greater classification accuracy. Studies confirmed that entropy ≥0.8 is related to at least 90% correct assignment, and considered adequate for the model^13^.

Second, after controlling for socio-demographic characteristics, a generalized linear Poisson model was used to explore the association between exposure clusters and birth defects. In Poisson regression, the expression relating these quantities is

3 $$\mathrm{log}(\mu )=({\beta }_{1}{X}_{1}+{\beta }_{2}{X}_{2}+\cdots +{\beta }_{k}{X}_{3})$$

Here, *µ* is the count of birth defects, and the regression coefficients ${\beta }_{1},{\beta }_{2},\ldots {\beta }_{k}$ are unknown parameters that need be estimated from the study data. Prevalence rate ratio (PRR) was used to indicate the magnitude of change, which would not be negative and <1 for a decrease and >1 for an increase. Then, the prevalence differences (PDs) in birth defects across different latent clusters were calculated. To evaluate the changing trends of differences in birth defects between different latent clusters over time, after adjusting for all possible confounding factors (ie, sociodemographic characteristics), the PDs across different latent clusters during 2009 to 2013 were obtained using a generalized linear model. Data was entered into Epidata 3.1 by double entry (CDC, Atlanta, GA, USA) and all statistical analysis was performed using Mplus version 5.1 (Linda Muthén & Bengt Muthén) and STATA version 12.0 software (STATA Corporation, College Station, TX, USA).

## Results

### Baseline characteristics of participants

In the study, only children who were alive at the time of sampling, were evaluated. Moreover, some participants were excluded from the study due to missing risk factors. As a result, a total of 9085 and 29094 young children and their mothers were enrolled in 2009 and 2013 respectively. The average age of children was 9.03 ± 5.03 months in 2009 and 16.88 ± 11.29 months in 2013 respectively (*P* < 0.001). Of the children, the proportion of boys was higher in 2009 than that in 2013 (55.96% and 54.35%, *P* < 0.05). The mean age of mothers was 25.88 ± 4.10 years and 28.03 ± 4.86 years respectively in 2009 and 2013 (*P* < 0.001). From 2009 to 2013, the percentage of mothers with educated beyond senior high school had increased from 25.52% to 38.25%. Based on tertiles of household wealth index, there was a different distribution of households in the poor, medium and rich category between 2009 and 2013. We also observed a higher proportion of participants from middle Shaanxi province in 2009 survey compared to 2013 survey. Amongst the mothers, the average parity differed significantly between 2009 and 2013. Any difference in residence during the pregnancy could not be observed in these two surveys. More detailed description of socio-demographic characteristics of participants was provided in Table 2. It was found that the rate of folic acid supplement was dramatically increased from 26.40% to 67.37% between 2009 and 2013. The proportion of exposure to risk factors among mothers including maternal illness, drug use, and adverse pregnancy outcomes consistently rose from 2009 to 2013, expect environmental risk factors and unhealthy lifestyles (Table 3).

**Table 2:** Baseline characteristics of participants between 2009 and 2013 in Shaanxi province*.

Socio-demographic characteristics	2009	2013
Parity^†^	1.22(0.43)	1.46(0.57)
Children age, mo^†^	9.03(5.03)	16.88(11.29)
Children gender^†^		
Male	5040(55.96)	15812(54.35)
Female	3967(44.04)	13281(45.65)
Maternal age, y^†^		
<25	4055(45.91)	7186(24.84)
25–29	3175(35.94)	12289(42.31)
≥30	1603(18.15)	9164(31.29)
Area^†^		
North	3472(38.22)	7476(25.69)
Middle	3234(35.60)	15695(53.95)
South	2379(26.19)	5923(20.36)
Maternal education level^†^		
No education	89(1.01)	560(1.93)
Primary school	927(10.56)	2965(10.20)
Junior high school	5522(62.91)	14408(49.62)
Senior high school	1398(15.93)	5811(20.01)
College and above	842(9.59)	5297(18.24)
Residence during the pregnancy		
Permanent	6741(87.55)	25640(88.12)
Floating	959(12.45)	3281(11.28)
HWI^†^		
Poor	1726(19.00)	12274(42.19)
Medium	4810(52.94)	5337(18.34)
Rich	2549(28.06)	11483(39.47)
Total	9085	29094

^*^Values are given as mean (SD) or the n (%) of the study population.

^†^Differences in socio-demographic characteristics between 2009 and 2013 were tested using t-test and χ^2^ tests.

**Table 3:** The rate of exposure to risk factors among mothers during pregnancy between 2009 and 2013 in Shaanxi Province^*^.

Risk factors	2009	2013
Folic acid supplementation^†^		
No	5303(73.60)	9380(32.63)
Yes	1902(26.40)	19369(67.37)
Maternal illness^†^		
No	4052(55.44)	14487(51.03)
Yes	3257(44.56)	13903(48.97)
Drug use^†^		
No	6501(88.03)	25185(84.68)
Yes	868(11.97)	4410(15.32)
Environmental risk factors^†^		
No	7226(98.05)	28468(98.55)
Yes	144(1.95)	420(1.45)
Adverse pregnancy outcomes^†^		
No	7732(90.53)	22653(80.61)
Yes	809(9.47)	5449(19.39)
Genetic factor		
No	7342(99.62)	28761(99.51)
Yes	28(0.38)	143(0.49)
Maternal unhealthy lifestyle^†^		
No	4930(66.34)	21028(72.79)
Yes	2501(33.66)	7862(27.21)

^*^Values are given as the n (%) of the study population.

^†^Differences in risk factors between 2009 and 2013 were tested using χ^2^ tests.

### Latent class cluster analysis

To determine the most optimal number of classes for our study, we began by reviewing the IC indices presented in Table 4. From 1-class model to 4-class model, we observed that BIC, AIC and SSA-BIC first rose in 2-class model and then sharply decreased in 3-class model and slightly increased in four-class model (Table 4). The various indices each consistently suggested the three-class model provides a significantly better fit, with the lowest BIC, AIC, and SSA-BIC. On review of the entropy values from one-class to four-class, all were more than 0.90, suggesting great classification accuracy. Although a four-class model might provide higher classification accuracy of the data compared to the three-class model, this additional class was composed of a relatively small number (proportionally, <1.0%) of participants. This, coupled with a preference for the AIC, BIC, SSA-BIC values, and parsimony, led us to choose a three-class model as the optimal model for further analysis in the study.

**Table 4:** Goodness of fit measures of four different class models*.

Model	LL	BIC	AIC	SSA-BIC	Entropy
1-cluster	−358685.72	717519.13	717399.43	717474.64	1.000
2-cluster	−394126.32	788518.77	788302.64	788439.32	0.924
**3-cluster**	**−187062.76**	**374442.03**	**374185.53**	**374346.69**	**0.971**
4-cluster	−209385.68	418847.35	419172.25	419051.49	0.981

^*^AIC, Akaike Information Criteria; BIC, Bayesian Information Criteria; SSA-BIC, Sample-size-adjusted (SSA)-BIC.

**Figure 1:**
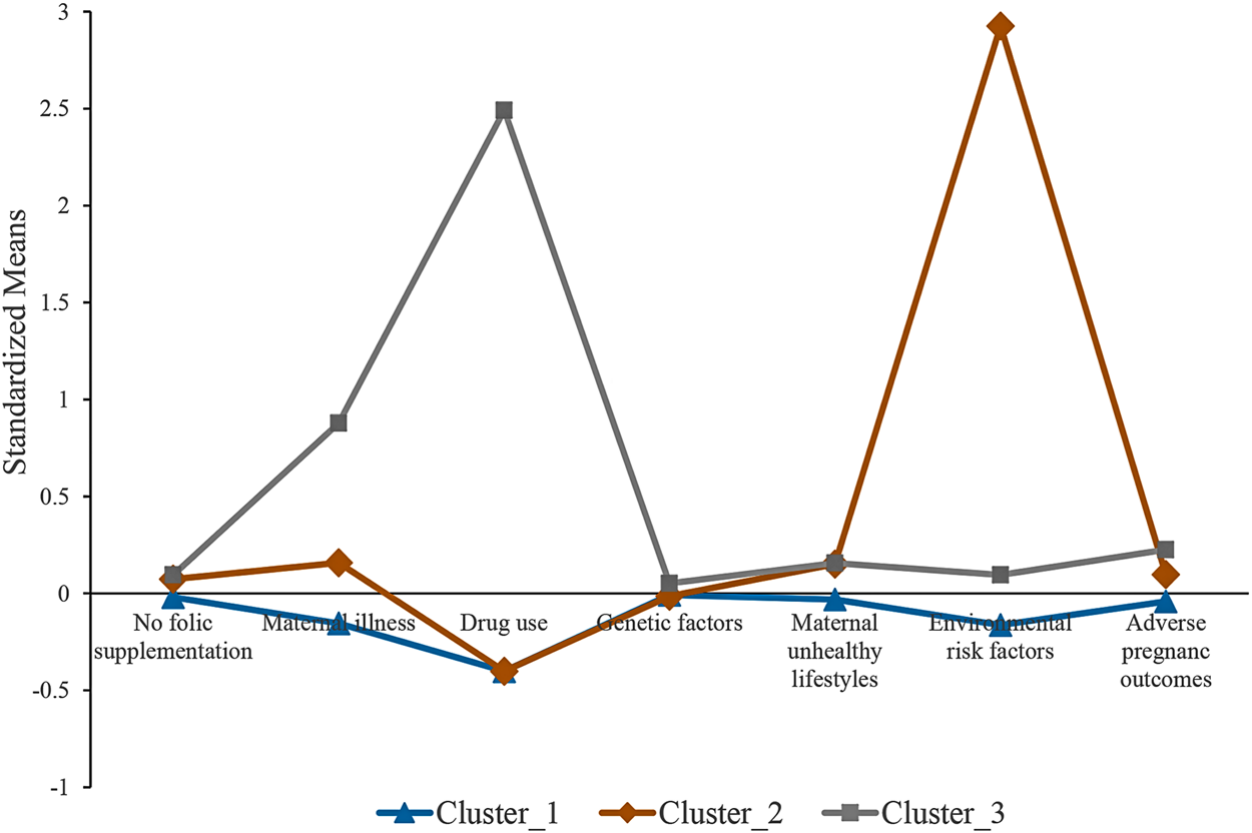
Standardized means of three clusters across the indicator variables. Cluster 1, 2, and 3 refer to the high-risk, moderate-risk and low-risk cluster, respectively

Figure 1 presented a clear depict of standardized means of seven indicators across three latent clusters. Cluster 3 had a 13.09% of participants (n = 4996) and the largest standardized means of most indicators, including not folic acid supplement, genetic factor, adverse pregnancy outcomes, and maternal illness during pregnancy, drugs use during pregnancy, and maternal unhealthy lifestyles during pregnancy. Therefore, the cluster 3 could be considered as high-risk cluster in which children were more likely to suffer from birth defects. A total of 32063 participants in cluster 1 accounted for the vast majority of the whole population (83.98%) in the study. Cluster 1 had the lowest standardized means of almost all seven indicators, and could be interpreted as low-risk cluster. Cluster 2 consisted of 1120 participants with a proportion of 2.93% in total, but comprised of the highest level of exposures to environmental risk factors during pregnancy and the second highest level of not folic acid supplement, genetic factor, adverse pregnancy outcomes, maternal illness during pregnancy and maternal unhealthy lifestyles during pregnancy, so was considered as moderate-risk cluster.

### Relationship between birth defects and three latent clusters

According to latent class cluster analysis based on the pooled data, the proportions from cluster-1 to cluster-3 in 2009 was 88.97%, 1.49%, 9.54% respectively, and similarly in 2013 were 82.42%, 3.39%, 14.19% respectively, between which we found a significant difference in the distribution of the three latent clusters (*P* < 0.001). To explore the impact of identified latent clusters on the birth defects in 2009 and 2013, a generalized linear Poisson model was employed to control for socio-demographic characteristics. Table 5 showed that with cluster-1 as the reference group, the prevalence ratio rate (PRR) of birth defects in cluster 3 was 1.93 (95%CI: 1.26, 2.95) in 2009 and 2.28 (95%CI: 1.91, 2.73) in 2013, but PRR in cluster 2 was not significant in both years. It was obvious in 2009 rather than 2013 that the higher the education level of mothers was, the PRR was lower. In 2013, higher economic level of mothers was associated with the lower risk for birth defects (PRR: 0.80, 95%CI: 0.67, 0.96), but the permanent residents were more likely to suffer from birth defects (PRR: 1.56, 95%CI: 1.26, 1.93). Moreover, the regional differences in the prevalence of birth defects were also found with higher prevalence in Middle and South Shaanxi than that in North Shaanxi in both 2009 and 2013.

**Table 5:** The association of birth defects with baseline characteristics between 2009 and 2013^*^.

Baseline characteristics	Any birth defects		Cardiovascular system malformation		Nervous system malformation	
	2009	2013	2009	2013	2009	2013
**Latent cluster of risk factors**						
Low risk cluster	Reference	Reference	Reference	Reference	Reference	Reference
Moderate risk cluster	2.02(0.74, 5.49)	1.43(0.96, 2.13)	2.71(0.36, 20.28)	**2.56(1.44, 4.57)**	—	—
High risk cluster	**1.93(1.26, 2.95)**	**2.28(1.91, 2.73)**	**2.99(1.28, 6.95)**	**3.14(2.35, 4.19)**	3.30(0.87,12.54)	1.06(0.36, 3.12)
**Parity**	1.04(0.68, 1.59)	**1.36(1.16, 1.60)**	0.66(0.23, 1.89)	**1.38(1.05, 1.81)**	0.67(0.16, 2.77)	1.47(0.71, 3.05)
**Children age, mo**	1.01(0.98, 1.04)	1.01(0.99, 1.02)	1.00(0.93, 1.07)	1.00(0.99, 1.01)	1.09(0.98, 1.20)	1.03(0.99, 1.07)
**Children gender**						
Male	Reference	Reference	Reference	Reference	Reference	Reference
Female	1.03(0.74, 1.43)	0.97(0.82, 1.14)	1.22(0.60, 2.50)	0.97(0.74, 1.29)	1.22(0.40, 3.73)	1.14(0.49, 2.63)
**Mother’s education**						
No education	Reference	Reference	Reference	Reference	Reference	Reference
Primary school	**0.27(0.11, 0.66)**	1.11(0.60, 2.04)	0.21(0.04, 1.10)	0.56(0.22, 1.39)	—	1.67(0.21, 13.41)
Junior high school	**0.23(0.10, 0.51)**	0.97(0.54, 1.74)	**0.11(0.02, 0.52)**	0.69(0.30, 1.61)	0.17(0.02, 1.55)	0.31(0.04, 2.61)
Senior high school	**0.17(0.07, 0.41)**	0.91(0.49, 1.69)	**0.09(0.02, 0.54)**	0.71(0.29, 1.74)	0.14(0.01, 1.81)	0.35(0.03, 3.47)
College and above	**0.13(0.05, 0.36)**	0.90(0.48, 1.70)	**0.12(0.02, 0.68)**	1.06(0.42, 2.63)	0.13(0.01, 1.60)	0.09(0.01, 1.60)
**Mother’s age, y**						
<25	Reference	Reference	Reference	Reference	Reference	Reference
25–29	1.09(0.74, 1.62)	0.92(0.74, 1.15)	1.28(0.57, 2.87)	1.09(0.74, 1.60)	3.52(0.85, 14.61)	2.20(0.61, 8.01)
≥30	0.87(0.51, 1.52)	0.93(0.73, 1.20)	0.58(0.14, 2.42)	1.26(0.82, 1.94)	4.30(0.67, 27.81)	0.80(0.18, 3.60)
**Wealth index**						
Poor	Reference	Reference	Reference	Reference	Reference	Reference
Medium	1.16(0.73, 1.84)	0.92(0.74, 1.15)	0.86(0.32, 2.34)	1.11(0.78, 1.59)	0.98(0.25, 3.98)	1.84(0.71, 4.75)
Rich	1.25(0.78, 1.99)	**0.80(0.67, 0.96)**	1.48(0.58, 3.80)	0.80(0.59, 1.10)	0.39(0.06, 2.44)	0.51(0.16, 1.63)
**Residence during the pregnancy**						
Floating	Reference	Reference	Reference	Reference	Reference	Reference
Permanent	1.20(0.76, 1.89)	**1.56(1.26, 1.93)**	2.06(0.86, 4.93)	**1.72(1.21, 2.43)**	2.60(0.55, 12.36)	0.37(0.05, 2.73)
**Area**						
North Shaanxi	Reference	Reference	Reference	Reference	Reference	Reference
Middle Shaanxi	**2.46(1.61,3.77)**	**1.68(1.33, 2.11)**	1.04(0.45, 2.43)	1.32(0.89, 1.97)	**0.12(0.03, 0.60)**	2.61(0.93, 7.28)
South Shaanxi	0.89(0.52, 1.52)	**1.81(1.40, 2.35)**	0.59(0.21, 1.65)	**2.54(1.67, 3.87)**	**0.14(0.03, 0.70)**	0.94(0.23, 3.86)

^*^Prevalence Rate Ratio (PRR) and 95% confidence interval are given to indicate the magnitude of change.

Further, we also investigated the relationship between cardiovascular system malformation and latent clusters. Compared to cluster 1, in these two surveys, the higher PRR of cardiovascular system malformation in cluster 3 was observed. The permanent residents from South Shaanxi were also important contributor of cardiovascular system malformation. Higher education level of mothers was correlated with the decreasing risk of cardiovascular system malformation in 2009. Nervous system malformation was also entered into the study, but any significant differences were not found among these socio-demographic characteristics except for survey areas.

### Prevalence of birth defects across identified latent clusters between 2009 and 2013

In our survey population, the prevalence of birth defects among young children had increased from 1.72% in 2009 to 2.16% in 2013, an increment rate of nearly 0.44%. With regard to nervous system malformation, the prevalence was decreasing from 0.14% to 0.09%, a descent rate of 0.05%. Especially for cardiovascular system malformation with a relatively high growth rate of 0.38%, the prevalence also had rose from 0.39% to 0.77% over the same time.

Further, we examined the differences in the prevalence of birth defects, cardiovascular system malformation, and nervous system malformation among three latent clusters between 2009 and 2013 (Table 6). It was clear that the rate of any birth defects and cardiovascular system malformation was higher in cluster 3 than that in cluster 1 in both two years. After adjusting for confounding factors using a generalized linear Poisson model, the results showed that in high-risk cluster the prevalence of nervous system malformation decreased by approximately 2.71%, and the occurrence rate of cardiovascular system malformation rose by 0.92% from 2009 to 2013. In high-risk cluster, however, the whole prevalence of birth defects had not been increased in the same period. In the meanwhile, the Prevalence differences (PDs) of birth defects and cardiovascular system malformation between cluster 3 and cluster 1 were increased by 0.60% and 0.85% respectively over the studied period. On the contrary, the prevalence difference in nervous system malformation between latent cluster 3 and cluster 1 reduced approximately 1.00% from 2009 to 2013, but it was not statistically significant.

**Table 6:** Prevalence of birth defects across different latent clusters from 2009 to 2013 *.

Latent clusters	2009	2013	Difference during 2009–2013,%
**Any birth defects**			
Low risk cluster (C1)	123(1.52)	429(1.79)	0.09(0.12)
Moderate risk cluster (C2)	4(2.96)	26(2.64)	−0.65(0.64)
High risk cluster (C3)	29(3.34)	174(4.21)	0.12(0.24)
**PD** ^**‡**^			
C1-C2	−1.44	−0.85	−0.26(0.24)
C2-C3	−0.38	−1.57	0.46(0.58)
C1-C3	−1.82^†^	−2.42^†^	0.60(0.30)^†^
**Nervous system malformation**			
Low risk cluster (C1)	10(0.12)	21(0.09)	−0.76(0.49)
Moderate risk cluster (C2)	0(0.0)	0(0.00)	—
High risk cluster (C3)	3(0.35)	4(0.10)	−2.71(1.48)^†^
**PD** ^**‡**^			
C1-C2	0.12	0.09	—
C2-C3	−0.35	−0.10	—
C1-C3	−0.23	−0.01	−1.00(0.85)
**Cardiovascular system malformation**			
Low risk cluster (C1)	26(0.32)	138(0.58)	0.57(0.25)
Moderate risk cluster (C2)	1(0.74)	13(1.32)	0.32(1.20)
High risk cluster (C3)	8(0.92)	74(1.79)	0.92(0.44)^†^
**PD** ^**‡**^			
C1-C2	−0.42	−0.74	0.50(0.70)
C2-C3	−0.18	−0.47	0.25(1.12)
C1-C3	−0.6^†^	−1.21^†^	0.85(0.42)^†^

*Prevalence differences in birth defects, nervous system malformation, cardiovascular system malformation across latent clusters between 2009 and 2013 were obtained using generalized linear Poisson model adjusting for baseline characteristics.

^†^P < 0.05. ^‡^PD = prevalence difference.

## Discussion

The population-based epidemiological survey was the largest sample study to cover all likely risk factors for birth defects in Shaanxi Province of Northwest China. Based on overall risk factors of pooled data in 2009 and 2013, three different latent clusters were extracted using LCCA to evaluate the synthetic effect of overall maternal exposures on birth defects. A generalized linear Poisson model was further used to evaluate the change in the prevalence of birth defects among latent clusters in Shaanxi province during 2009–2013. In high risk cluster, in particular, the prevalence of cardiovascular system malformation progressively increased over time, and the rate of nervous system malformation reduced in the same period.

First, our results confirmed that the prevalence of the whole birth defects, cardiovascular system malformation and nervous system malformation in high risk cluster were the highest among the three latent clusters. Compared with the low-risk cluster, it was found that women in the high-risk cluster were nearly twice more likely to have offspring with birth defects after adjusting for maternal socio-demographic variables. In the meanwhile, the likelihood of cardiovascular system malformation in the high-risk cluster was three times than that of low-risk cluster. This could be due to the fact that the high risk cluster comprised of the highest value of almost all risk factors, such as not folic acid supplement, genetic factor, adverse pregnancy outcomes, and maternal illness during pregnancy, drugs use during pregnancy, and maternal unhealthy lifestyles during pregnancy. It suggested that the strongest synthetic effects of all possible risk factors for birth defects had been observed among high-risk cluster. To screen high-risk groups of expectant mothers who live in high-risk areas using LCCA will be useful for future government-led, integrated interventions for birth defects.

Second, the changes in the prevalence of cardiovascular system malformation and nervous system malformation in high risk cluster in Shaanxi province during 2009–2013 were obvious after adjusting for socio-demographic factors using the generalized linear Poisson model. In the high risk cluster, the prevalence of cardiovascular system malformation was increased by approximately 0.92% from 2009 to 2013, but the rate of nervous system malformation was reduced by around 2.71% in the same period. The bulk of epidemiological evidence suggested that have long been linked to folic acid supplementation^14–16^. In our study, a small proportion of mothers (28.05%) received periconceptual folate in high-risk cluster in 2009, but this percentage increased to 70.89% in the same cluster in 2013. This can largely contribute to the reduction of nervous system malformation among children in high risk cluster. However, we also observe the rate of exposure to other risk factors such as maternal medical history, drug use, previous adverse pregnancy outcomes, and unhealthy lifestyles among mothers, have risen during 2009–2013, which could explain in part the increment of cardiovascular system malformation for a 5-year period. Further studies will be required to corroborate these findings and, if confirmed, to elucidate possible reasons for these changes.

Third, after controlling for socio-demographic characteristics using generalized linear Poisson model, the disparities in the rates of birth defects and cardiovascular system malformation between low risk cluster and high risk cluster were increasingly expanded during 2009–2013. This results showed the birth defects might more concentrated in the high-risk cluster over time rather than low risk or medium risk cluster. It could conclude that compared to low-risk cluster, the synthetic effects of all possible risk factors on birth defects were more strengthened among high-risk cluster in surveyed areas over time. Therefore, it was necessary to pay more attentions to those in the high risk cluster in terms of implementing interventions targeting behavior changes and environmental risk factors control.

Furthermore, socio-demographic factors have been found to play an important role in the presentation of birth defects. Maternal education has also been implicated in the incidence of birth defects^17, 18^. In our study, similarly, a lower percentage of birth defects or cardiovascular system malformation was found among women with high education level compared to those without education. This could be one of the reasons that highly educated mothers are more likely to have more knowledge of prenatal care, higher awareness regarding periconceptual supplementation with folate, and abstention from certain environmental risk factors that plays a major role in the incidence. Subjects with the highest household SES index had the lowest risks of birth defects, which is consistent with the previous studies^19–21^. In this study, higher maternal parity was also associated with birth defects and cardiovascular system malformation. Due to reproductive organ damage and dysfunction caused by an excessive number of parturitions^22^, it was inferred that it may increase the risk of birth defects. For example, a slightly larger rate of birth defects among infants born to mothers with three or more prior births than those born to first-time mothers was showed in Texas study between 1999 and 2003^23^. There was a significantly lower rate of birth defects and cardiovascular system malformation in floating populations than that in permanent populations, which was consistent with our previous study^24^. In this study, we also observe a significant regional difference in the rate of birth defects. For example, a higher prevalence of birth defects and cardiovascular system malformation in south and central Shaanxi in contrast with that in south Shaanxi.

The benefit of this LCCA is that it gives us the ability to assess the impact of groups of maternal exposures not in isolation but in patterns or groups, as they commonly existed. Based on LCCA, however, previous researches more focused on the association between dietary patterns and birth defects, including CHDs, spina bifida, cleft lip, and hypospadias^25–30^. To date, there are only two published researches that used LCCA to explore the synergistic effects of multiple risk factors and to screen high-risk groups in a high-risk birth defects area. In a cross-sectional study, Cao *et al*. employed the LCCA to identify maternal exposure clusters, and explore the association between clusters of risk factors and birth defects^31^. Similarly in another cross-sectional study, Zhu *et al*. also introduced the latent class model to examine differences in how factor profiles and single factors are related to birth defects^32^. However, our study has combined the two surveys for pooled data and extended the LCCA to evaluate the trend in the prevalence of birth defects among latent clusters over time.

Further, another strengths of LCCA is that allows adjustment for covariates, quantification of the uncertainty of class membership, and assessment of goodness of fit. The use of maternal exposure factors as continuous variables enable the study to avoid some loss of information of categorizing the data in previous study^32^. Finally, associations between latent clusters and birth defects were adjusted for socio-demographic covariates, which were possibly associated with latent clusters. However, we have to acknowledge some limitations needed to address in the study. Frist, due to the cross-sectional design of the study, the causal relationships between environmental risk factors and birth defects were not confirmed, and the results should be explained cautiously. Second, the identified latent clusters may be subjected to unavailable information on mothers’ diet, which might influence the generalizability of our study. Third, the research lack of data on related genes that was highly associated with the increasing risk of birth defects in the context of genotype–phenotype associations. Despite these limitations, this study has still provided important information on the synergistic effects of multiple risk factors on the birth defects in Northwest China, and filled a gap in temporal trend of the synergistic effects of birth defects among different risk clusters.

In conclusion, our study introduced LCCA to evaluate the synthetic effects of the risk factors related to birth defects and investigate the temporal trend of the synthetic effects of these factors from 2009 to 2013. According to latent class cluster analysis based on the pooled data, the population was classified into three different latent clusters, including high-risk cluster, medium-risk cluster and low-risk cluster. The synthetic effects of maternal exposure to multiple risk factors could increase the risk of overall birth defects and cardiovascular system malformation among live births, and this risk is significantly higher in high-risk cluster than that in low-risk cluster. In high risk cluster, the prevalence of cardiovascular system malformation progressively increased over time, but the rate of nervous system malformation reduced in the same period. The primary prevention of birth defects targeting combined behavioral change and environmental risk control, should be provided for women of reproductive age in study area, particularly for high-risk cluster with the greatest needs.
